# Post-Operative Pain

**Published:** 1913-10

**Authors:** William F. Waugh

**Affiliations:** Chicago, Ill.


### Post-Operative Pain.

BY WILLIAM F. WAUGH, M. D., CHICAGO, ILL.

The other day I had a tooth extracted. It was broken off and for ten minutes the operator hunted for the root, unsuccessfully. The gas anesthesia endured about 50 seconds, so that I suffered acutely for over nine minutes. I reached my home, enduring agonies; every tooth in both jaws aching and exquisitely tender. I consulted the dentist, who assured me the pain was from the operative injury.

That gave me the clue—one of the advantages claimed for H-M-C anesthesia is the abolition or rather prevention of post-operative pain—and I am a firm believer in preventive medicine. If this remedy prevents such pain it also relieves it, so I took hypodermatically a half-tablet containing morphine, grain 1-8, hyoscine, grain 1-200, and cactoid, grain 1-128.

In a few minutes the pain seemed bearable; in twenty minutes it was fairly controlled. I slept well that night. Next day the face was swollen, but the pain presented only in occasional twinges.

Judging from the reports of correspondents I had learned to look on this combination as the most powerful means at our disposal for the severer forms of pain; but this was my first personal experience with it.

In view of the atrocious character of the pain I am amazed at the speedy and permanent relief afforded by this minute dose. I had sometimes taken a dose of morphine alone to break up a cold, with good effect indeed, but a miserable headache next morning that always made me regret I had not let the cold run its course. This time there was no unpleasant symptoms following, not even constipation.

